# Interspecific Niche Competition Increases Morphological Diversity in Multi-Species Microbial Communities

**DOI:** 10.3389/fmicb.2021.699190

**Published:** 2021-07-30

**Authors:** Xiao-Lin Chu, Quan-Guo Zhang, Angus Buckling, Meaghan Castledine

**Affiliations:** ^1^College of Life and Environmental Sciences, Environment and Sustainability Institute, University of Exeter, Penryn, Cornwall, United Kingdom; ^2^State Key Laboratory of Earth Surface Processes and Resource Ecology and MOE Key Laboratory for Biodiversity Science and Ecological Engineering, Beijing Normal University, Beijing, China

**Keywords:** competition, niche, community ecology, adaptive radiation, microbial ecology, evolutionary ecology

## Abstract

Intraspecific competition for limited niches has been recognized as a driving force for adaptive radiation, but results for the role of interspecific competition have been mixed. Here, we report the adaptive diversification of the model bacteria *Pseudomonas fluorescens* in the presence of different numbers and combinations of four competing bacterial species. Increasing the diversity of competitive community increased the morphological diversity of focal species, which is caused by impeding the domination of a single morphotype. Specifically, this pattern was driven by more diverse communities being more likely to contain key species that occupy the same niche as otherwise competitively superior morphotype, and thus preventing competitive exclusion within the focal species. Our results suggest that sympatric adaptive radiation is driven by the presence or absence of niche-specific competitors.

## Introduction

Adaptive radiation is a key component of biodiversity generation (Dieckmann and Doebeli, 1999; Schluter, 2000; Losos, 2010; Kassen, 2014). Population diversification – the precursor to adaptive radiation – not only depends on genetic characteristics of a population, such as cryptic genetic variation (Zheng et al., 2019) and mutation/recombination rate (Gavrilets and Vose, 2005; Lanfear et al., 2010), but also on abiotic and biotic environmental conditions (Grant, 1986; Rainey and Travisano, 1998; Buckling and Rainey, 2002; Hall and Colegrave, 2007; Meyer and Kassen, 2007; Betts et al., 2018). Intraspecific competition, in particular, is suggested to be a key driver of diversifying selection in a wide range of taxa (Schluter, 2000; MacLean et al., 2005; Svanback and Bolnick, 2007). However, the role of interspecific competition is less clear. Interspecific competitors might contribute to increased diversification by creating new ecological niches or could select for different resource usage to that of coexisting species (Emerson and Kolm, 2005; Svanback and Bolnick, 2007; Erwin, 2008; Calcagno et al., 2017). On the other hand, interspecific competitors may inhibit diversification due to fewer vacant niches or reduced population sizes (Gómez and Buckling, 2013; Ghoul and Mitri, 2016; Schluter and Pennell, 2017; Pontarp and Petchey, 2018; Harvey et al., 2020). Moreover, the neutral theory of community assembly proposes that all species are functionally equivalent for community assembly and maintenance (Hubbell, 2001; Harris et al., 2017), implying a neutral role of interspecific competition in diversification.

Direct experimental tests of the role of interspecific competitors on diversification have typically used the bacterium *Pseudomonas fluorescens* as the focal species because of its propensity to morphologically diversify over 10s of generations. These studies have generated contrasting results. Gómez and Buckling (2013) demonstrated lower evolved diversity in resource use of an initially isogenic population of *P. fluorescens* in the presence vs. absence of the natural microbial community in soil; Jousset et al. (2016) reported that increasing the number of competing *P. fluorescens* isolates resulted in an increased diversifying selection of the focal strain; while Zhang et al. (2012) reported little effect of a competitor (*Pseudomonas putida*) on *P. fluorescens* diversification.

We argue that the inconsistent results could be reconciled based on the theoretical framework emerging from biodiversity-ecosystem functioning studies (Schulze and Mooney, 1993; Tilman, 1997; Loreau et al., 2001). Specifically, more diverse competitors may generally reduce the diversification of focal species due to the reduced niche availability and total abundance of a focal species, which is akin to a complementarity effect. Meanwhile, a sampling effect may also take place, as a species-richer competitor community is more likely to contain particular species that can strongly affect the focal species. However, its effect on the diversification within the latter may depend on the specific competitive interactions, either positive (if it competes with a dominant genotype and thus prevent competitive exclusion within the focal species) or negative (if it competes with all genotypes and strongly reduces the total abundance of the focal species). While the Jousset study created a diversity gradient, all the competitors were *P. fluorescens* isolates, limiting the potential for resident niche occupation (Jousset et al., 2016). A recent study of natural microbial communities from the Earth Microbiome Project found a unimodal relationship between diversity and diversification: community diversity beget diversity in low-diversity biomes and reach plateaus when niches are increasingly filled in high-diversity biomes (Madi et al., 2020).

To further investigate the role of interspecific diversity on diversification, we evolved *P. fluorescens* SBW25 across a diversity gradient of a synthetic microbial community isolated from potting compost, which consists of four species that can stably coexist in a relatively oligotrophic medium when incubated in the lab (Castledine et al., 2020). *Pseudomonas fluorescens* can rapidly diversify into three main colony morphotypes when propagated in spatially heterogeneous microcosms (Rainey and Travisano, 1998). These morphotypes can be typically identified as: ancestor-like smooth (SM) that occupies the liquid phase, wrinkly spreader (WS), which arises by spontaneous mutations and forms a self-supporting mat on the air-liquid interface, and fuzzy spreader (FS), which occupies the anaerobic niche (Rainey and Travisano, 1998). The driver of diversification has been shown to be competition for oxygen and other nutrients (Koza et al., 2011; Kassen, 2014). The present study measured the morphological diversification of *P. fluorescens* in the presence of microbial communities, in which a diversity gradient was set-up using four species, to determine the diversity of competing microbial communities on adaptive diversification.

## Materials and Methods

### Experimental Evolution

**Figure 1:**
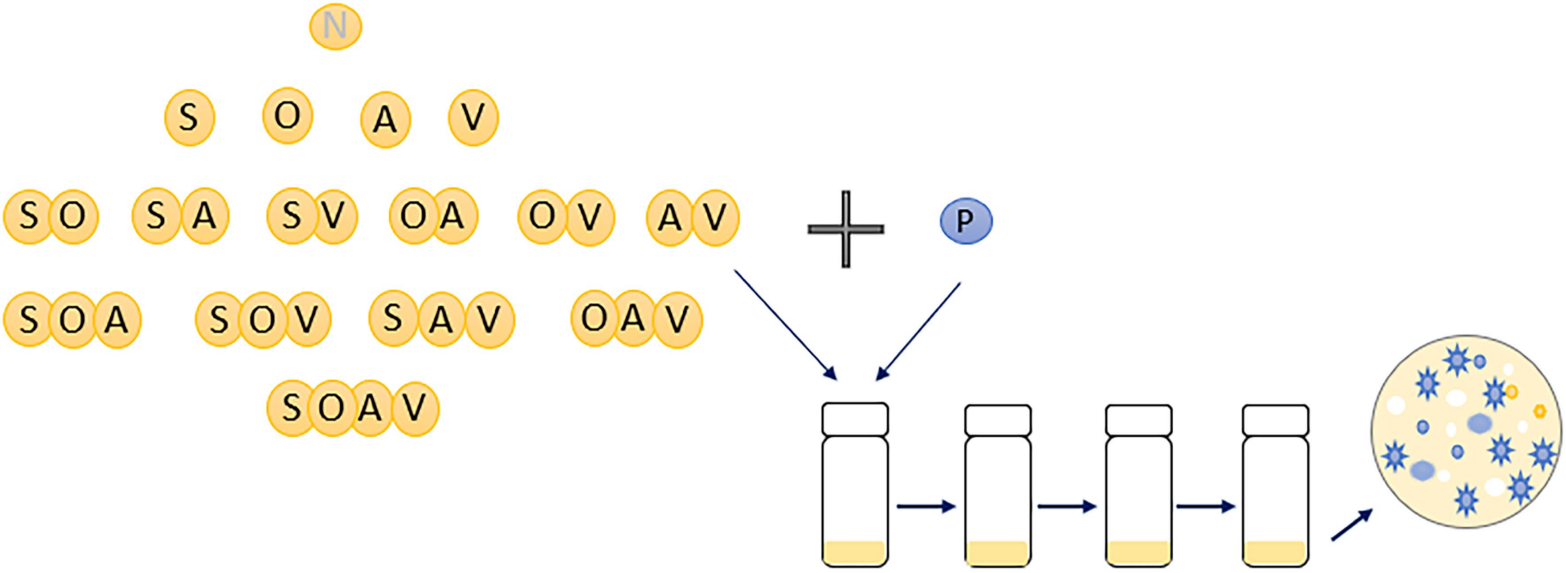
An illustration of experimental design of the present study. Sixteen different microbial communities were set-up with species diversity ranging from 1 (monoculture of *Pseudomonas fluorescens*) to 5 (with all five species). Six replicates were conducted for each community combination. N represents no other species were inoculated into the microcosms; P, A, O, S, and V represents *Pseudomonas fluorescens*, *Achromobacter*, *Ochrobactrum*, *Stenotrophomonas* and *Variovorax*, respectively. Diversity of the focal species, *Pseudomonas fluorescens*, at the end of the evolution experiment was estimated based on its morphological diversity of 100 randomly chosen colonies (blue ones).

Communities were set up with a SM clone of *P. fluorescens* SBW25 with isogenic clones of *Achromobacter* sp. (A), *Ochrobactrum* sp. (O), *Stenotrophomonas* sp. (S), and *Variovorax* sp. (V), which can be distinguished by their unique colony morphologies (Supplementary Figure 1A; Castledine et al., 2020; Padfield et al., 2020 unpublished). Every community combination of the four species with *P. fluorescens* was set up across all the levels of diversity with six replicates for each community combination. Therefore, a total of 16 different communities were established with total species richness ranging from 1 (only *P. fluorescens*) to 5 (all five species). Specifically, there were one 1-species community treatment, four 2-species treatments, six 3-species treatments, four 4-species treatments, and one 5-species treatment (Figure 1). Communities were grown in 25 ml glass vials with loosened lids with 6 ml of M9KB media (glycerol 10 g L^−1^, proteose peptone no.3 20 g L^−1^, KH_2_PO_4_ 3 g L^−1^, NaCl 0.5 g L^−1^, and NH_4_Cl 1 g L^−1^). Prior to experimental set-up, each species was grown for 2 days in M9KB media at 28°C to achieve high-cell densities. Cell densities of each species were diluted into M9 buffer to approximately 10^4^ CFUs uL^−1^. Each microcosm was inoculated with 20 uL per species (~10^5^ cells) and incubated at 28°C in static, after which 60uL was transferred every 7 days for a total of three transfers. Culture samples were cryogenically frozen at −80°C in 50% glycerol (final concentration: 25%) at each transfer. Species densities within each microcosm at each transfer were determined by plating culture dilutions onto KB agar and counting the number of colony-forming units (CFUs) after 2 days of incubation at 28°C.

### Measurement of Diversity

Community diversity was estimated by species richness (the number of species). The ancestral smooth *P. fluorescens* diversified into three morphotypes (SM, WS, and FS; Supplementary Figure 1B) at the end point and the sympatric diversity of *P. fluorescens* populations was obtained by measuring the morphologies of 100 randomly chosen colonies (Buckling et al., 2000) and calculated as Simpson’s index: $\left(\frac{N}{N-1}\right)\left(1-\lambda \right)$, where *N* is the total number of colonies sampled from the focal population and $\lambda =1-\sum p_{i}{}^{2}$ ($p_{i}$ is the frequency of the *i*th morphotype; Simpson, 1949).

### Statistical Analysis

All analysis was conducted using R (version 3.5.2; R Core Team, 2018), and all plots were made using the R package “*ggplot2*” (Wickham, 2016). Simpson’s index was calculated using the package “*vegan*” (Oksanen et al., 2019). A linear model was used to analyze the relationship between *P. fluorescens* diversity and the proportion of WS morphotype; the relationship between inoculated density of the competing community and proportion of WS or *P. fluorescens* diversity; and the relationship between the initial or final density of *P. fluorescens* and its sympatric diversity. ANOVA was performed to analyze whether the presence or absence of competitors affected *P. fluorescens* diversity and the proportion of WS. Density data were log-transformed [log_10_ (1 + CFUs ml^−1^)].

The effect of community richness on the diversity of *P. fluorescens* were analyzed by an analysis of variance with sequential sum of squares (type I) using linear models (Schmid et al., 2002; Buzhdygan et al., 2020). To assess whether diversity effects were only due to the presence of single species, we fitted each species (presence/absence) before species richness in separate sequential analyses. If fitting single genotypes before species richness removed the effect of richness indices, the observed diversity effects may have been mainly caused by a sampling effect (the inclusion of a particular genotype in the community; Huston, 1997). To further explain whether species composition matters, we used ANOVA to test the effect of the presence of each species and their interactions on *P. fluorescens* diversity. The simplest model was obtained by AICc ranking using the R package “*MuMIn*” (Barton, 2020). The effects of the presence of single species and initial community diversity on the proportion of WS, and on the density of WS were tested with sequential linear models as described above.

## Results

### Dominant WS Morphotype Inhibits *Pseudomonas fluorescens* Diversity

**Figure 2:**
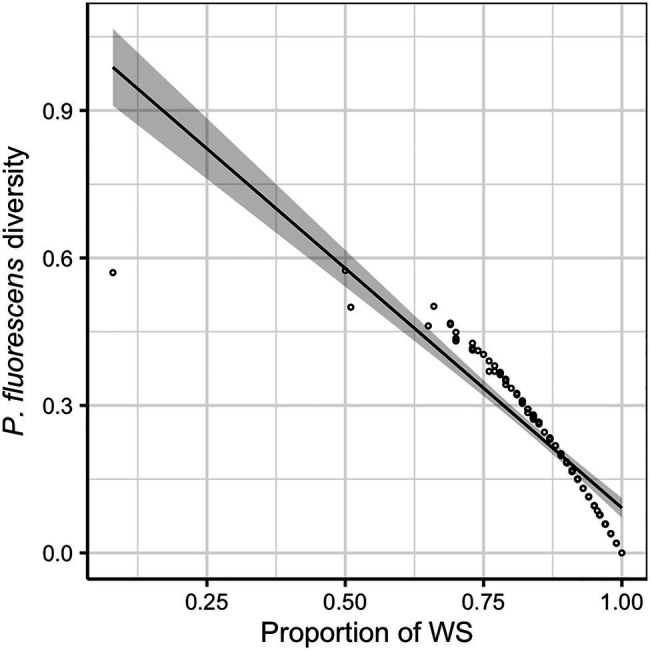
*Pseudomonas fluorescens* diversity is negatively correlated with the proportion of wrinkly spreader (WS). The regression line represents a significant linear relationship and shaded areas around lines show the 95% confidence intervals: y=1.066−0.974x, *F_1,94_* = 369.820, *p* < 0.001, adjusted *R^2^* = 0.795.

Across all evolution lines, the WS morphotype was found to dominate the diversified *P. fluorescens* populations (mean ± SD of the proportion of WS in *P. fluorescens* populations: 0.847 ± 0.128). A negative relationship was found between *P. fluorescens* diversity and proportion of WS (*F_1,94_* = 369.82, *p* < 0.001; Figure 2), indicating that *P. fluorescens* diversity is caused by reduced proportions of WS morphotypes. This implies that factors limiting the dominant WS morphotype would increase the sympatric diversity of *P. fluorescens*. Although the presence of competing communities was not found to affect *P. fluorescens* diversity (*F_1,94_* = 3.606, *p* = 0.061) or the proportion of WS (*F_1,94_* = 2.521, *p* = 0.116), the density of inoculated competing communities reduced the proportion of WS (*F_1,88_* = 5.346, *p* = 0.023; Supplementary Figure 2). In addition, a positive relationship was observed between the density of inoculated competing communities and the diversity of focal species (*F_1,88_* = 10.169, *p* = 0.002; Supplementary Figure 2), suggesting a competing effect of the microbial communities on *P. fluorescens*.

### Species-Specific Effects on *Pseudomonas fluorescens* Morphotype Diversity

**Figure 3:**
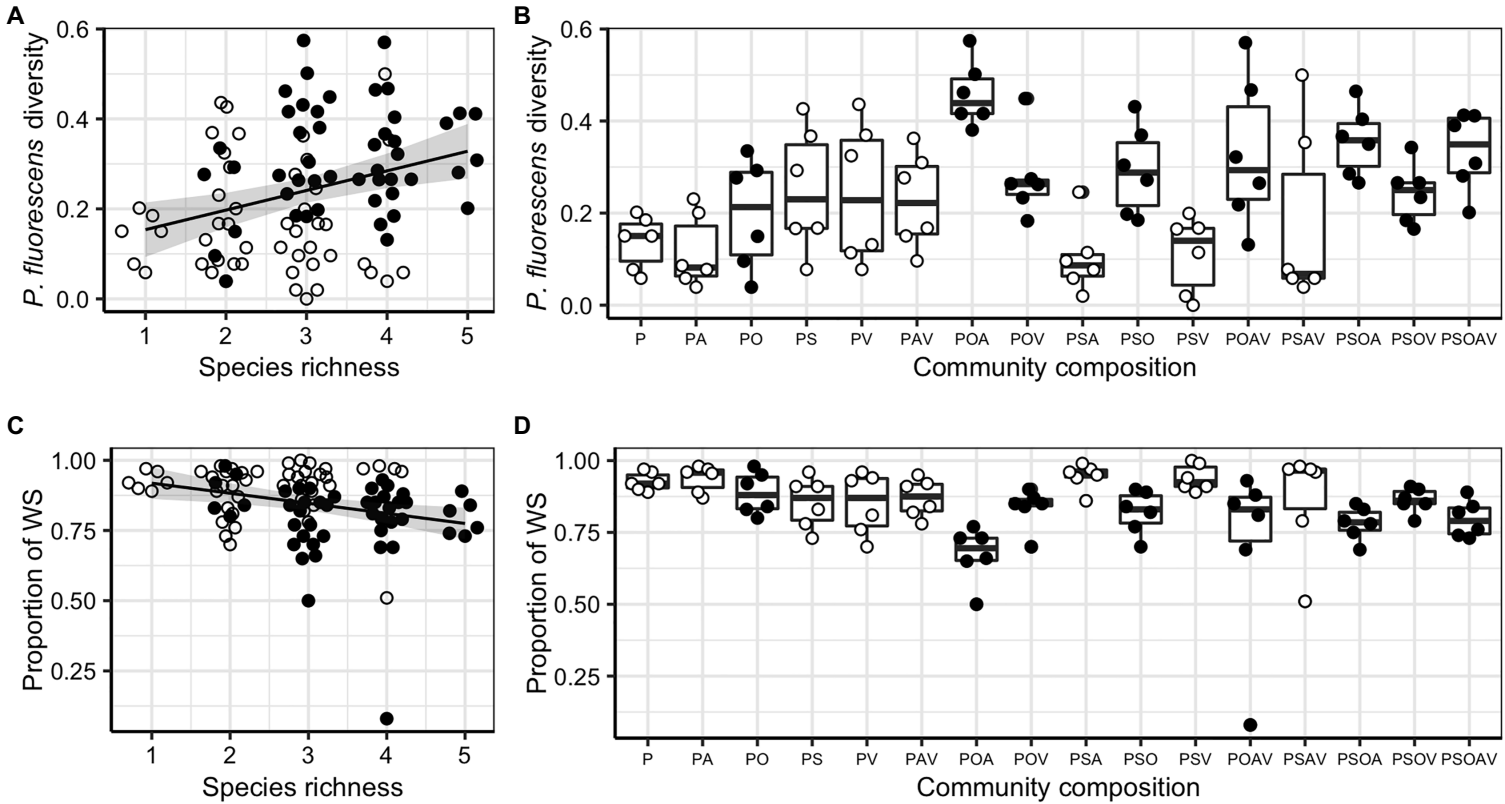
The effect of species richness, the presence of *Ochrobactrum* sp. (O) and community composition on *Pseudomonas fluorescens* diversity **(A,B)** and the proportion of wrinkly spreader (WS; **C,D**). For both responsive variables, regression lines showed significant linear relationships with species richness: y=0.110+0.044x, *F_1,94_* = 369.820, *p* < 0.001, adjusted *R^2^* = 0.088 **(A)**; and y=0.954−0.036x, *F_1,94_* = 8.061, *p* = 0.006, adjusted *R^2^* = 0.069 **(B)**. Shaded areas around lines show the 95% confidence intervals. Treatments containing O (filled dots) and not containing O (open dots) are highlighted to show the sampling effect of O. Tops and bottoms of the bars represent the 75th and 25th percentiles of the data, the middle lines are the medians, and the whiskers extend from their respective hinge to the smallest or largest value no further than 1.5 × interquartile range.

*Pseudomonas fluorescens* diversity was positively affected by increasing species richness (*F_1,94_* = 10.204, *p* = 0.002). However, the significant effect of species richness was removed when fitted after the presence/absence of the *Ochrobactrum* species (Figures 3A,B; Table 1), indicating that the observed diversity effect is mainly due to a sampling effect. Further analysis revealed that the diversity of *P. fluorescens* was also affected by the interaction between *Ochrobactrum* and *Achromobacter* species (*F_1,92_* = 9.537, *p* = 0.003). Qualitatively similar results were obtained using alternative community diversity metric (Simpson’s index; see Supplementary Text).

**Table 1:** Analysis of variance table of *F*-values on the effects of presence of specific species, and species richness on the diversity of *Pseudomonas fluorescens* populations.

Factor	*df*	*Achromobacter* sp.		*Ochrobactrum* sp.		*Stenotrophomonas* sp.		*Variovorax* sp.	
		*F*	*P*	*F*	*P*	*F*	*P*	*F*	*P*
Species presence	1	2.558	0.113	31.898	**<0.001**	0.293	0.590	0.027	0.871
Species richness	1	7.538	**0.007**	0.620	0.433	16.849	**<0.001**	13.174	**<0.001**
Residuals	93								

*The bold values indicate significant effects*.

While species *Achromobacter* (A), *Ochrobactrum* (O), *Stenotrophomonas* (S), and *Variovorax* (V) stably coexisted in a previous study, the presence of *P. fluorescens* destabilized this community in many combinations (Supplementary Figure 3). V was driven extinct in at least 3/6 replicates in 7/8 species combinations with V going extinct in 2/6 replicates in the PSAV evolution line. Similarly, S went extinct in 4/6 replicates in evolution lines PSO and PSOA, and in 1/6 replicates in evolution lines PSOV and PSV. These extinctions resulted in variable community diversities and the *P. fluorescens* final densities ranging from 4.40 × 10^8^ to 2.78 × 10^9^. These results indicate that O and A (to a less extent) are the main competitors of *P. fluorescens* and affect its diversity.

### Potential Mechanisms

We first tested if the patterns of diversification could be affected by *P. fluorescens* density by affecting the supply of mutations and the strength of diversifying selection. Neither inoculated *P. fluorescens* density nor its final density affected diversification. However, the final population size of *P. fluorescens* decreased in the presence of competing species (*F_1,94_* = 13.410, *p* < 0.001), suggesting the interspecific resource competition during the diversification of focus species. As A and O coexisted with *P. fluorescens*, while S and V went extinct in many combinations (Supplementary Figure 3), they are presumably the main competitors of *P. fluorescens*.

Given that *P. fluorescens* diversity is caused by the reduced proportion of WS, the increased diversity of focal species in the presence of O, and O and A together may be a result of competition with WS. Therefore, we tested whether the presence of particular species affected diversity of focal species by affecting the proportion of WS (the sampling effect). Although the proportion of WS was affected by species richness (*F_1,94_* = 8.061, *p* = 0.006), its effect was removed when fitted after the presence of O (Figures 3C,D; Table 2). In addition, a marginal sampling effect of A was also detected (Table 2). These results supported the competition between WS and O (and to a lesser extent A) in driving the diversification of *P. fluorescens*. As WS forms mats to occupy the air-liquid interface, we hypothesized that O (and A to a lesser extent) may compete with WS in this niche, reducing invasion of WS. O, A, and WS were found to form mats in media when grown in monoculture while V formed very thin mats and mats were absent for S and SM. The presence of O and A were, indeed, found to affect the density of WS (Supplementary Text; Supplementary Figure 4; and Supplementary Table 1), suggesting the niche competition of O and A with the derived WS morphs of *P. fluorescens*.

**Table 2:** Analysis of variance table of *F*-values on the effects of presence of specific species, and species richness on the proportion of wrinkly spreader.

Factor	*df*	*Achromobacter* sp.		*Ochrobactrum* sp.		*Stenotrophomonas* sp.		*Variovorax* sp.	
		*F*	*P*	*F*	*P*	*F*	*P*	*F*	*P*
Species presence	1	3.868	0.052	18.257	**<0.001**	0.807	0.371	0.249	0.619
Species richness	1	4.548	**0.036**	0.942	0.334	15.272	**<0.001**	8.955	**0.004**
Residuals	93								

*The bold values indicate significant effects*.

## Discussion

Here, we demonstrated that the colony morphological diversity of evolving *P. fluorescens* populations increased with the number of bacterial taxa it was co-cultured with. This pattern was primarily driven by the presence of a specific species (O) in more diverse communities, although there were also some interactive effects with other species. *Pseudomonas fluorescens* diversity was negatively correlated with the proportion of evolved WS morphotypes, which had a mean frequency of ~0.8 across all replicates. Most of the remaining *P. fluorescens* populations were SM morphotypes. Diversity was therefore maximized when the evolved WS less successfully dominating populations.

The presence of O (and to a lesser extent A) impeded the dominance of WS. The other species (S and V) were frequently driven extinct by the end of experiment, and hence would have imposed relatively little competition. We also observed that O and A produced more detectable mats (or biofilms) than the other two species, and mat formation is characteristic of WS [we tried to quantify the biofilms with the resazurin assay, (Peeters et al., 2008) but failed as their mats are not as definable as the biofilms of *P. fluorescens*]. This suggests that O and A, which are the strongest competitors in the community, are competing more with WS than SM by occupying a similar ecological niche to WS (Kim et al., 2014). Similar constraints on the invasion of evolved WS have been observed when the resident *P. fluorescens* WS genotypes were present in the populations (Brockhurst et al., 2007; Flohr et al., 2013).

The formation of biofilms and inhibiting the biofilm formation of other microbial populations could be an evolved competitive strategy since these genotypes could gain preferential access to resources and the biofilms could protect cells from environmental hazards (An et al., 2006; Stacy et al., 2016). For example, it has been reported that *P. aeruginosa* could prevent *Agrobacterium* from forming its own biofilm by surface blanketing (An et al., 2006), and *E. coli* can reduce the biofilm formation of Gram-positive and Gram-negative bacteria by producing extracellular polysaccharides or surfactants (Valle et al., 2006; Rendueles et al., 2011).

WS density was highly variable in the presence of O and A, and this may be explained by the advantage of forming biofilms and historical contingences. The diversified WS is a cooperating group that can enable individuals to align with them by overproducing an adhesive polymer and promote the colonization of the air-liquid interface though the overproduction is costly to individuals (Spiers et al., 2002; Rainey and Rainey, 2003). Therefore, if mutations to form WS arose late in a population or *P. fluorescens* initially grew slowly, WS may lose the chance to spread because the available niche is already partially occupied by competitor species.

It has been previously shown that the *Pseudomonas* sp. can stably coexist with the other four competing species (Castledine et al., 2020), while S and V were at low densities or went extinct in many combinations by the end of our experiment. Although a different *Pseudomonas* was involved here, it is not suggested to be a main cause of the instability as family-level bacteria are found to have similar function (Goldford et al., 2018; Louca et al., 2018). Therefore, the instability may be a result of different media used in the present studies that altered community composition (Goldberg and Miller, 1990; Goldford et al., 2018). Specifically, the rich media used here may have increased the likelihood of competitive exclusion by favoring the fast-growing *Pseudomonas*.

Limitations of the present study are noteworthy. The relative short-term evolution limited the study to further investigate more nuanced processes such as how within-species diversification feedback to affect community structure. Though the three morphotypes of *P. fluorescens* has been widely used for diversity estimation, less consideration has been given to its genetic diversity (Spiers et al., 2002; Fukami et al., 2007).

Diversity-dependent adaptive radiation theory predicts that more diverse communities are more likely to experience evolutionary diversification (Calcagno et al., 2017). This is not supported by our study despite a greater diversity of focal species in the presence of more diverse competitors was observed. Our study is instead consistent with previous work, suggesting that evolutionary processes are impeded by the presence of competitors (Hall et al., 2018; Scheuerl et al., 2020). In this case, competitors reduced the dominance of the evolved WS genotypes, most likely because of shared niche occupation in spatially structured (static) microcosms. Though the reduced population size of focal species by interspecific competition may affect the adaptive radiation process, *P. fluorescens* is less affected in our working system as the focal species showed an advantage and dominated the microbial communities after three transfers (Johansson, 2008; Osmond and de Mazancourt, 2012; Zhao et al., 2018). Our results may help to understand the inconsistent effects of interspecific competitors on diversification of resident species across different studies and suggest that the presence of niche-specific competitors is a possible explanation.

## Data Availability Statement

The datasets presented in this study can be found in online repositories. The names of the repository/repositories and accession number(s) can be found at: https://doi.org/10.6084/m9.figshare.14471229.

## Author Contributions

All authors contributed to the design of the study. X-LC and MC conducted the experiments. All authors contributed to the data analysis and the writing of the manuscript.

## Conflict of Interest

The authors declare that the research was conducted in the absence of any commercial or financial relationships that could be construed as a potential conflict of interest.

## Publisher’s Note

All claims expressed in this article are solely those of the authors and do not necessarily represent those of their affiliated organizations, or those of the publisher, the editors and the reviewers. Any product that may be evaluated in this article, or claim that may be made by its manufacturer, is not guaranteed or endorsed by the publisher.

## Abbreviations

A: *Achromobacter* sp.
O: *Ochrobactrum* sp.
S: *Stenotrophomonas* sp.
V: *Variovorax* sp.
SM: Smooth morph
WS: Wrinkly spreaders
FS: Fuzzy spreader
