# Supplementary material for: Interspecific Niche Competition Increases Morphological Diversity in Multi-Species Microbial Communities
Source: Front Microbiol. 2021 Jul 30;12:699190. doi: 10.3389/fmicb.2021.699190 (PMC8362326; doi:10.3389/fmicb.2021.699190)
Supplement: Supplementary file 1 [file Data_Sheet_1.DOCX]

Supplementary Material

# Supplementary Data

The effect of inoculated community diversity was also estimated when calculated as the Simpson’s index: $(\frac{N}{N-1})(1-\lambda)$ , where *N* is the total number of individuals sampled from the community and $\lambda= 1- \sum{p_{i}}^{2}$ ($p_{i}$ is the frequency of the *i*th species). The role of community diversity on *P. fluorescens* diversification was assessed with sequential sum of squares using linear models and the sampling effect of each species was also analyzed. Though community diversity affected *P. fluorescens* diversity (*F_1,94_* = 5.380, *P* = 0.023), its effect was removed when fitted after the presence of O (*F_1,93_* = 1.937, *P* = 0.167) and, to a lesser extent, the presence of A (*F_1,93_* = 3.317, *P* = 0.072; Supplementary Figure 5; Supplementary Table 2). This suggests a sampling effect of O and to a lesser extent A in driving the diversification of the focal species instead of the effect of community diversity.

The density of WS decreased with the number of bacterial taxa it was co-cultured with (*F_1,94_* = 13.592, *P* < 0.001). In addition, the presence of A and O both affected the density of WS (Supplementary Figure 5; Supplementary Table 1).

## Supplementary Figures


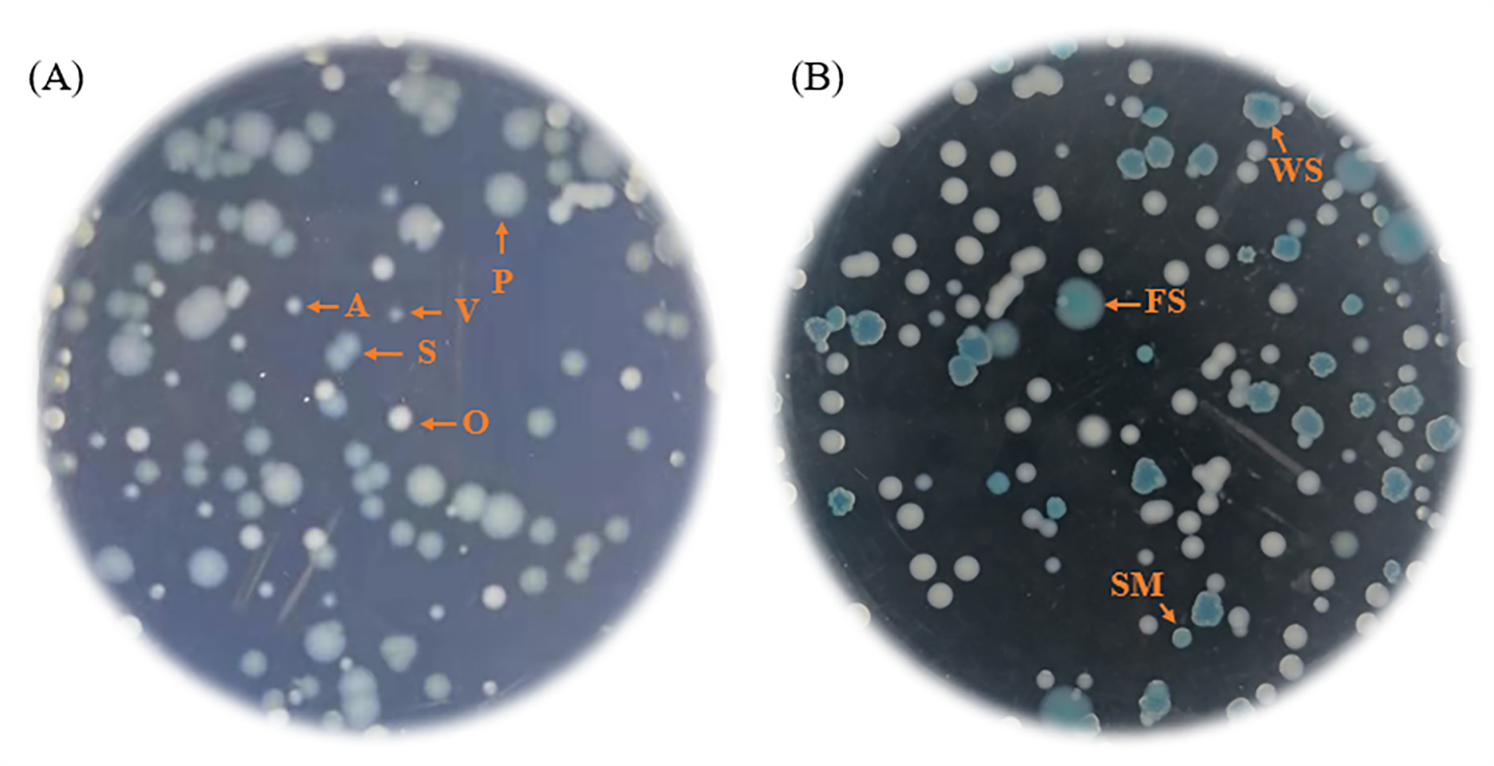


**Supplementary Figure 1.** Colony morphologies of the five species (A) and three diversified morphologies of *Pseudomonas fluorescens* (B). Figure (a) shows the colony morphologies of *P. fluorescens* SBW25 *lacZ* (P), *Achromobacter* sp. (A), *Ochrobactrum* sp. (O), *Stenotrophomonas* sp. (S) and *Variovorax* sp. (V) on KB agar plate. Figure (b) illustrates the three morphotypes of diversified *P. fluorescens* SBW25 *lacZ* (SM: ancestor-like smooth; WS: wrinkly spreader; and FS: fuzzy spreader) on KB agar plates with X-gal.


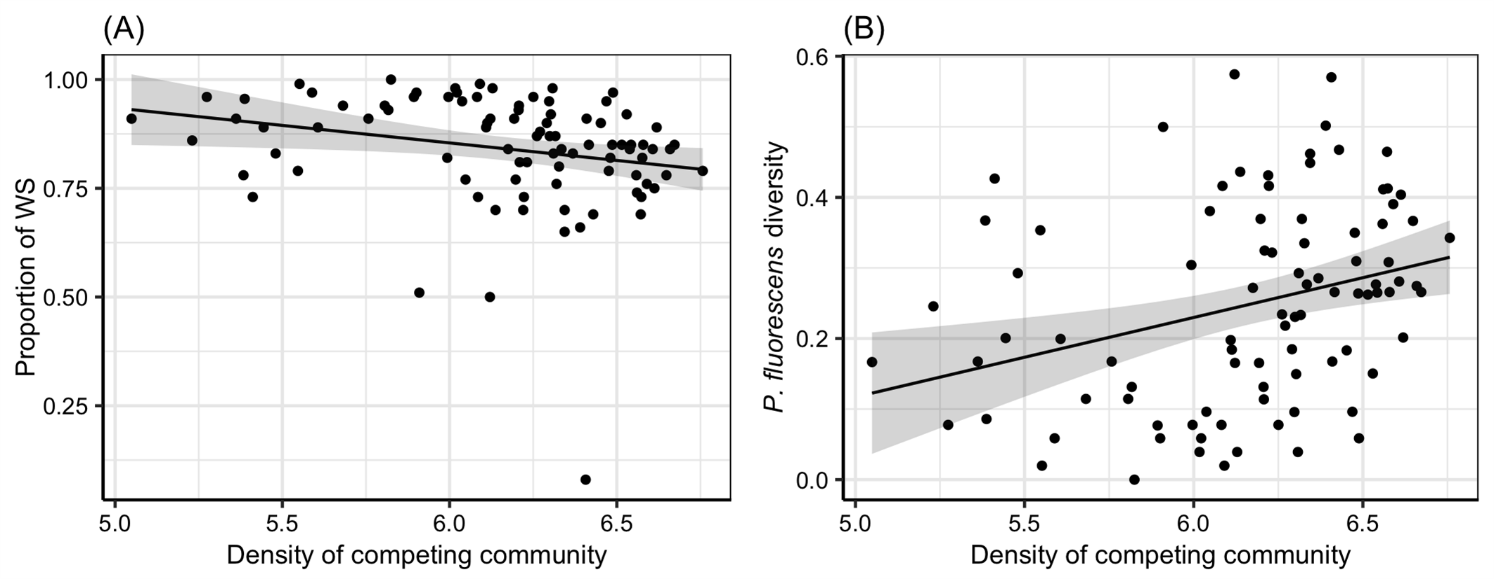


**Supplementary Figure 2.** The effect of inoculated density of competing communities on (a) the proportion of wrinkly spreader (WS) and (b) *P. fluorescens* diversity. The density of competing communities were log-transformed [log_10_ (1 + CFUs mL^-1^)]. Lines represent significant linear regressions and shaded areas around lines show the 95% confidence intervals: $\text{y}\text{ }\text{=}\text{ }\text{1.367}\text{ }\text{-}\text{ }\text{0.085x}$, *F_1,88_* = 5.346, *P* = 0.023, adjusted *R^2^* =0.047 (a); and $\text{y}\text{ }\text{=}\text{ }\text{0.123}\text{ }\text{-}\text{ }\text{0.517x}$, *F_1,88_* = 10.169, *P* = 0.002, adjusted *R^2^* =0.093 (b).


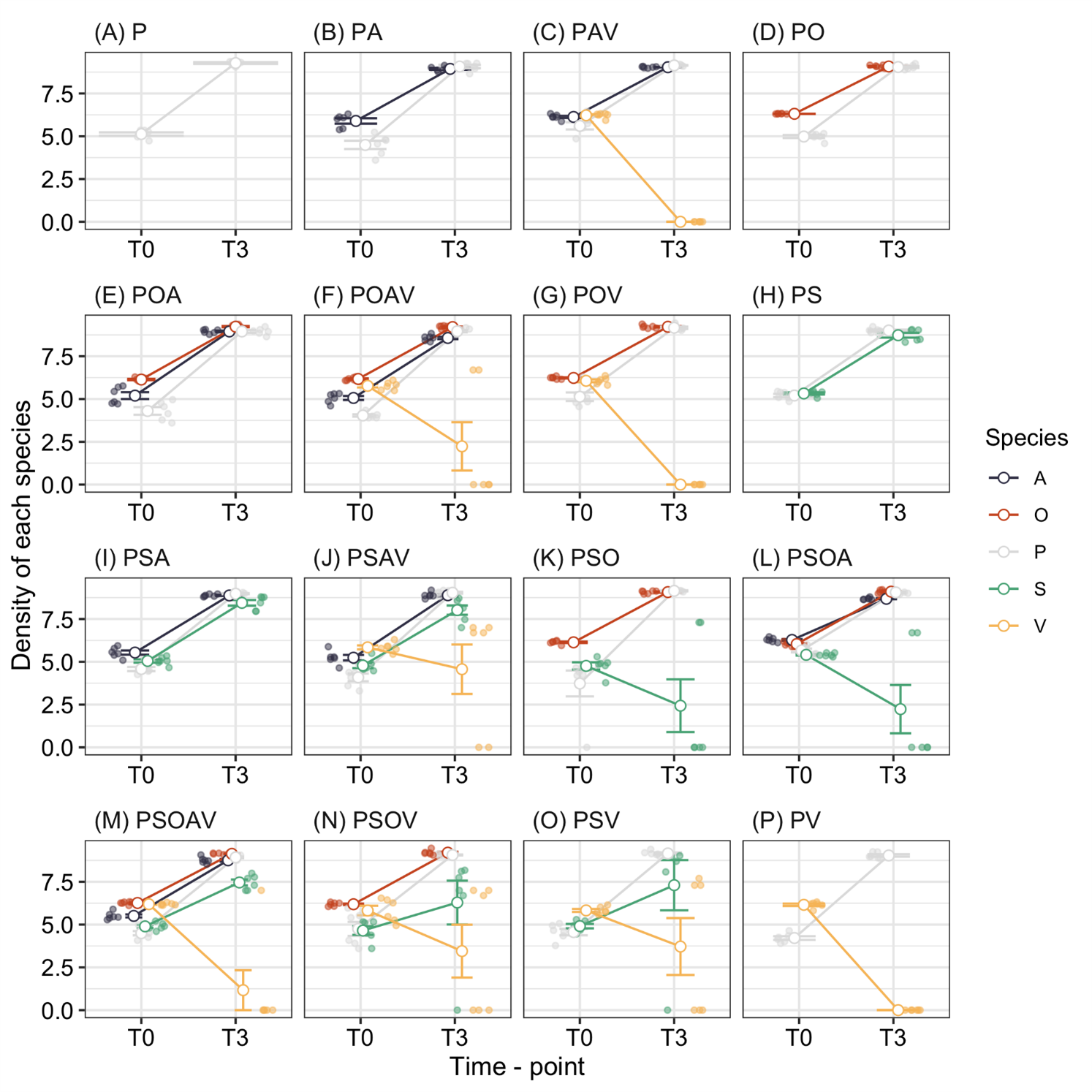


**Supplementary Figure 3.** Changes in the density of each species from the start (T0) to the end (T3) of the experiment across each community combination. Large points indicate means while smaller points indicate individual treatment replicates. Bars = ±SE.


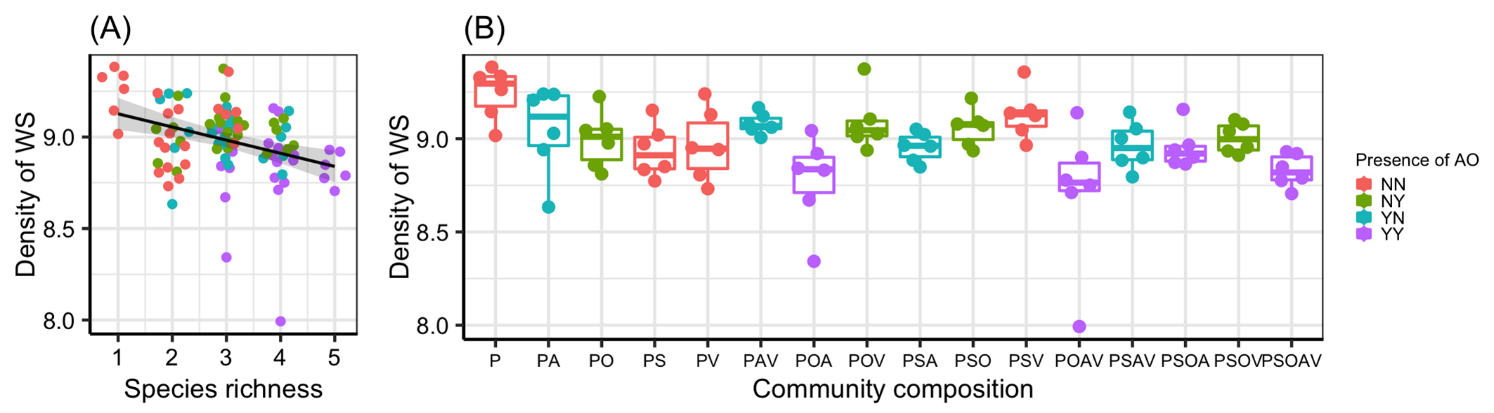


**Supplementary Figure 4.** The effect of species richness, the presence of *Ochrobactrum* sp. (O) and *Achromobacter* sp. (A), and community composition on the density of wrinkly spreader (WS). Density of WS was log-transformed [log_10_ (1 + CFUs mL^-1^)]. The thick line shows a significant linear regression between the density of WS and species richness and shaded areas around lines show the 95% confidence intervals: $\text{y}\text{ }\text{=}\text{ }\text{9.199}\text{ }\text{-}\text{ }\text{0.072x}$, *F_1,94_* = 13.592, *P* < 0.001, adjusted *R^2^* =0.117. The density of WS was also affected by the presence of O and A; and red, green, blue and purple points or boxes represent the communities not containing O or A, not containing A but O, containing A but not O, and containing both A and O, respectively. Tops and bottoms of the bars represent the 75th and 25th percentiles of the data, the middle lines are the medians, and the whiskers extend from their respective hinge to the smallest or largest value no further than 1.5 × interquartile range.


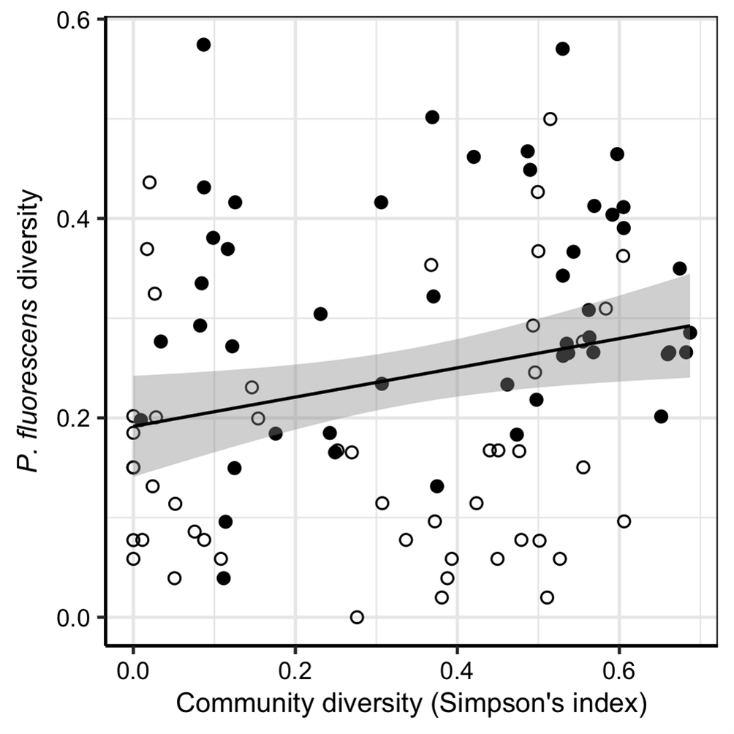


**Supplementary Figure 5.** The effect of community diversity (Simpson’s index) on *P. fluorescens* diversity. The regression line shows a significant effect and shaded areas around lines show the 95% confidence intervals: $\text{y}\text{ }\text{=}\text{ }\text{0.192}\text{ }\text{+}\text{ }\text{0.147x}$, *F_1,94_* = 5.380, *P* = 0.023, adjusted *R^2^* =0.044. The filled dots show plots containing O and the open dots show plots without O.

**Supplementary Table 1.** Analysis of variance table of *F*-values on the effects of presence of specific species, and species richness on the density of wrinkly spreader.

| Factor | df | *Achromobacter* sp. | | *Ochrobactrum* sp. | | *Stenotrophomonas* sp. | | *Variovorax* sp. | |
| --- | --- | --- | --- | --- | --- | --- | --- | --- | --- |
|  |  | *F* | *P* | *F* | *P* | *F* | *P* | *F* | *P* |
| Species presence | 1 | 13.090 | **< 0.001** | 8.610 | **0.004** | 0.076 | 0.784 | 0.384 | 0.537 |
| Species richness | 1 | 5.004 | **0.028** | 6.635 | **0.012** | 17.237 | **< 0.001** | 15.388 | **< 0.001** |
| Residuals | 93 |  |  |  |  |  |  |  |  |

**Supplementary Table 2.** Analysis of variance table of *F*-values on the effects of presence of specific species, and inoculated community diversity (Simpson’s index) on the diversity of *P. fluorescens* populations.

| Factor | df | *Achromobacter* sp. | | *Ochrobactrum* sp. | | *Stenotrophomonas* sp. | | *Variovora*x sp. | |
| --- | --- | --- | --- | --- | --- | --- | --- | --- | --- |
|  |  | *F* | *P* | *F* | *P* | *F* | *P* | *F* | *P* |
| Species presence | 1 | 2.450 | 0.121 | 32.347 | **< 0.001** | 0.269 | 0.605 | 0.025 | 0.875 |
| Inoculated community diversity | 1 | 3.317 | 0.072 | 1.937 | 0.167 | 7.753 | **0.006** | 5.727 | **0.019** |
| Residuals | 93 |  |  |  |  |  |  |  |  |
